# Versatile chip-based nanoscopy becomes ready for histopathology assessment

**DOI:** 10.1038/s41377-022-00781-0

**Published:** 2022-04-07

**Authors:** Martin Lopez-Garcia

**Affiliations:** Natural and Artificial Photonic Structures Group, International Iberian Nanotechnology Laboratory, Braga, 4715-330 Portugal

**Keywords:** Nanophotonics and plasmonics, Super-resolution microscopy, Other photonics

## Abstract

Nanoscopy is a mature technology used routinely in life science to obtain images well below the optical diffraction limit. But the use of nanoscopy in histopathology assessment is very limited mostly due to the low throughput of traditional nanoscopic techniques. So far, Chip-nanoscopy, nanoscopy in which sample illumination is performed by an integrated photonic chip instead of bulk optics, has been shown to provide an enhanced field of view and throughput for cell biology. Now, a new development shows that chip-nanoscopy also offers interesting progress for the study of histological samples offering a complementary technique to electron microscopy for histopathology assessment.

## Abstract (web summary)

A recent publication shows that chip-nanoscopy is a suitable technique for histopathological assessment. This development opens the door to the clinical implementation of optical nanoscopy in tissue damage assessment.

## Graphical abstract

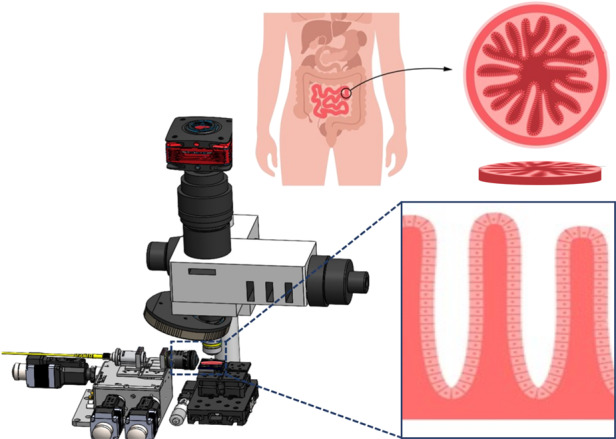

It has been less than 30 year since the first realizations of optical nanoscopy, optical microscopy with resolutions well below the diffraction limit. Since the first demonstrations by Hell^1^, Betzig^2^ and Moerner^3^ that granted them the Nobel prize in Chemistry 2014, the field has progressed extremely rapidly into a well-established technology implemented and used routinely in microscopy facilitates worldwide. The possibility to image cellular processes with resolutions in the order of tens of nm has revolutionized cell biology and, in combination with other nanoscopy techniques such as cryo-electron microscopy, is allowing us to understand cellular and molecular processes at a detail unimaginable only 15 years ago. Yet, the most common optical nanoscopy techniques require costly and bulky equipment which usually hampers its deployment beyond well-equipped and funded laboratories. In addition, optical nanoscopy still suffers from low throughput in its implementations.

Probably the most widespread approach to optical nanoscopy is single-molecule localization microscopy (SMLM) such as stochastic optical reconstruction microscopy (dSTORM)^4^. But the large number of images of the same area necessary to generate the final image strongly reduces the throughput and field of view. Other techniques such as stimulated emission depletion microscopy (STED)^1^ and structured illumination microscopy (SIM)^5^ are based on the generation of complex illumination patterns that usually require from advanced optical components with a very precise alignment and costly implementations. Moreover, the throughput over a large field of view, although an improvement against SMLM, is also low due to the requirement to scan an excitation beam through large areas in very short steps and long image reconstruction processes. These critical bottlenecks are important for applications in life science where samples are often only suitable for inspection during a short period of time or where large fields of views to image several cells are required.

So-called chip-nanoscopy was recently realized by Diekmann et al.^6^, through creative implementation. They showed that the common glass slide substrate could be substituted by an optical chip that allows the generation of illumination patterns suitable for optical nanoscopy. Sample illumination using waveguides is a widespread technique since Granding et al propose its first implementations^7^ although always under diffraction-limited conditions. The approach by Diekmann and colleagues used high refractive index materials which together with broad strip waveguide designs makes it possible to obtain a high evanescent field enabling chip-based nanoscopy techniques such as e.g., dSTORM. Moreover, the wide waveguide design allows to oscillate the coupling of the excitation hence producing illumination pattern variations which make the technique also suitable for SLML implementations^6^. More recently, it was also shown that similar designs allow even structured illumination chip-based SIM (cSIM)^8^.

Histopathology, the diagnosis through the study of tissue diseases is one of those fields were nanoscopy is of course very attractive given the wide range of diseases that can only be diagnosed through tissue visualizations below the diffraction limit. But the low throughput of most nanoscopy techniques has prevented their implantation for histopathology. Recently, important progress in this direction has been reported by E. Villegas-Hernández et al.^9^. They demonstrated for the first time, the use of chip-based nanoscopy for histopathological assessment.

The work by Villegas-Hernández and colleagues builds upon chip-nanoscopy techniques (Fig. 1) to introduce important progress in their implementation for histopathology^9^. The use of a chip allows the authors first to test histological samples with diffraction-limited techniques like Total Internal reflection fluorescence (TIRF) microscopy. Although diffraction-limited, this is a very interesting step because the chip provides an enhanced FOV with scalable magnification just upon change of the imaging lens. The authors demonstrate the viability with a relevant clinical case prepared by standard labeling and ultra-microtome techniques demonstrating the suitability of the technique for tissue analysis in clinical environments. But the most important advance of this work is on the demonstration of several super-resolution techniques for histopathology with the same chip.

**Fig. 1:**
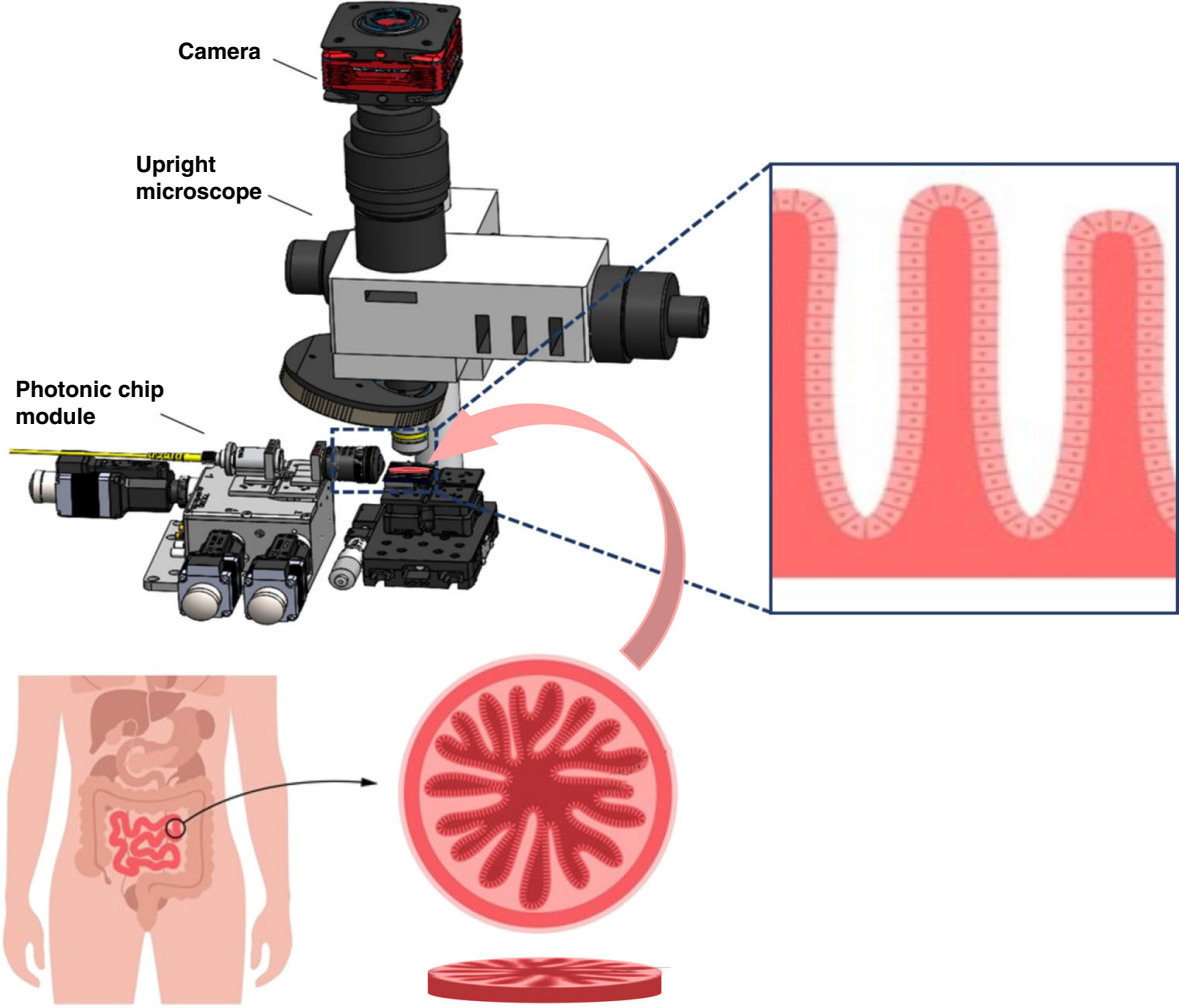
Schematics of chip-based nanoscopy for histopathology. Diagram of chip-based nanoscopy setup extracted from the publication by Villegas-Herandez et al.^9^ and sketch of a histology sample for intestine tissue for illustration purposes

Very interestingly the authors show the successful implementation of dSTORM in thin sections of tissue. But perhaps even more relevant is their demonstration of intensity fluctuation techniques (IFON). Using IFON, the chip based-implementation largely outperforms the throughput and field of view reported before with standard nanoscopy approaches to histology^10^. A comparison suggests a reduction from 2.5 h to 10 min for image acquisition in similar conditions. And although comparisons will be highly dependent on technique, type of sample and sample preparation it is clear that the chip-based nanoscopy could open the road for the use of optical microscopy as a substitute to cryo-electron microscopy in histology. In fact, the work by E. Villegas-Hernández and colleagues show an implementation for correlative light and electron microscopy. This “cherry on the top of the cake” demonstrates the great potential of this technique. The chip allows simultaneously optical nanoscopy with common microscopes and ultrastructural analysis with electron microscopy which opens the doors to the identification of specific components within the damaged tissues.

Chip-based nanoscopy has been shown to be a cost-effective and high through output alternative to standard optical super-resolution techniques. With the extension of its possibilities to histology, it could become a relevant tool in clinical analysis in the near future allowing for a faster and easier diagnosis. For this, it is still necessary to define standards like chip designs, coupling strategies, sample preparation protocols as well as the production of user-friendly implementations. The consistency of the results shown in the work highlighted here is very important progress in this direction.
